# *Lespedezadanxiaensis* (Fabaceae), a new species from Guangdong, China, based on molecular and morphological data

**DOI:** 10.3897/phytokeys.185.72788

**Published:** 2021-11-15

**Authors:** Wan-Yi Zhao, Kai-Wen Jiang*, Zai-Xiong Chen, Bin Tian, Qiang Fan

**Affiliations:** 1 State Key Laboratory of Biocontrol and Guangdong Provincial Key Laboratory of Plant Resources, School of Life Sciences, Sun Yat-sen University, Guangzhou 510275, China Sun Yat-sen University Guangzhou China; 2 Key Laboratory of Biodiversity Conservation in Southwest China, National Forestry and Grassland Administration, Southwest Forestry University, Kunming 650224, China Southwest Forestry University Kunming China; 3 Ningbo Botanical Garden, Ningbo 315201, China Ningbo Botanical Garden Ningbo China; 4 Administrative Commission of Danxiashan National Park, Shaoguan 512300, China Administrative Commission of Danxiashan National Park Shaoguan China

**Keywords:** Danxia landform, Guangdong, Leguminosae, new species, taxonomy

## Abstract

*Lespedezadanxiaensis* (Fabaceae), a new species from Danxiashan National Nature Reserve in Guangdong Province, is described and illustrated. The new species is morphologically similar to *Lespedezapilosa*, but it can be easily distinguished by its thin leathery leaflets and long peduncles. Phylogenetic analysis based on ITS confirmed that the new species belongs to Lespedezasubg.Macrolespedeza. The new species is the first known species of *Lespedeza* endemic to Danxia landform and is currently only known from Mount Danxia, Guangdong.

## Introduction

*Lespedeza* Michx. (Fabaceae) is a member of the subtribe Lespedezinae (Hutch.) Schub. of the tribe Desmodieae (Benth.) Hutch. The genus is characterised by shrubs, sub-shrubs or perennial herbs with tri-foliolate leaves (Huang et al. 2010; Ohashi and Nemoto 2014). *Lespedeza* has a disjunct distribution being present in both East Asia and North America, and consists of 46 species including the recently described *L.pseudomaximowiczii* D. P. Jin, Bo Xu bis & B. H. Choi and *L.hengduanshanensis* (C.J. Chen) Bo Xu bis, X.F. Gao & Li Bing Zhang (Ohashi and Nemoto 2014; Xu et al. 2014; Jin et al. 2018). The genus is traditionally divided into two subgenera, viz. Lespedezasubg.Lespedeza and L.subg.Macrolespedeza (Maxim.) H. Ohashi, based on the presence or absence of cleistogamous flowers (Ohashi 1982; Huang et al. 2010; Xu et al. 2012). Molecular phylogenetic studies, using nrITS and five chloroplast fragments (*rpl*16, *rpl*32-*trn*L, *rps*16-*trn*Q, *trn*L-F and *trn*K/*mat*K), showed that subg. Lespedeza is paraphyletic since the North America taxa (belonging to L.subg.Lespedeza) are sister to East Asia taxa that included members of both subgenera (Xu et al. 2012). Based on these results, Ohashi and Nemoto (2014) re-circumscribed both subgenera and confined L.subg.Lespedeza to North America, while L.subg.Macrolespedeza was confined to Asia.

During a botanical expedition to Danxiashan National Nature Reserve, Renhua County, Guangdong Province from May to October, 2020, we discovered an unknown species of *Lespedeza*. It is similar to *L.pilosa* (Thunb.) Siebold & Zucc. in indumentum (densely villous throughout), procumbent stems and ovate to obovate leaflets, but differs from the latter by its leathery leaflets, pinkish corolla and longer peduncles of chasmogamous flowers. After carefully checking specimens and literature, together with a molecular phylogenetic analysis based on Internal Transcribed Spacers (ITS), we demonstrated it is indeed a new species; thus here, we describe and illustrate it.

## Materials and methods

### Morphological study

The morphological characters were examined, based on the living plants and specimens kept in the herbaria **IBSC**, **NPH**, **SWFC** and **SYS**, herbarium acronyms as in Thiers (2021).

### Taxon sampling and molecular analyses

**Figure 1.**
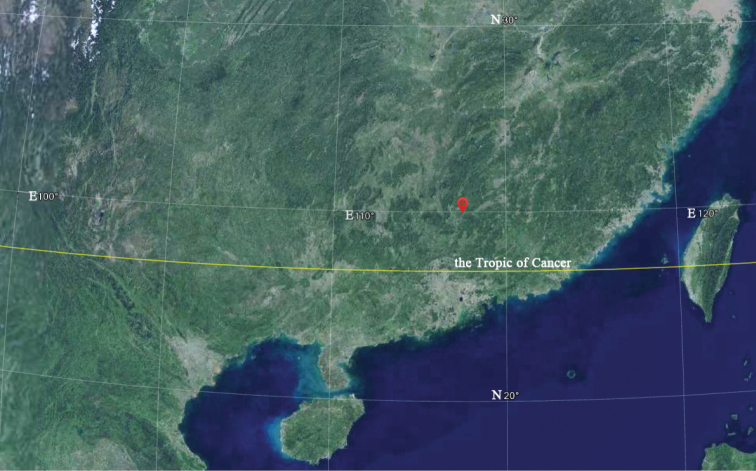
Satellite image for the location of *Lespedezadanxiaensis* Q. Fan, W.Y. Zhao & K.W. Jiang.

Three individuals of *L.danxiaensis* were collected from Danxiashan National Park, Guangdong province, China from July to September in 2020 (Fig. 1). Voucher specimens were deposited in the Herbarium of Sun Yat-sen University (SYS). The nuclear DNA Internal Transcribed Spacers (ITS) was used for reconstructing the phylogeny of the new species and its related taxa (Xu et al. 2012). A total of 45 accessions, representing 33 species of *Lespedeza* [including two nominal species viz. *L.nipponica* Nakai and *L.japonica* L. H. Bailey, which had been synonymised with *L.formosa* (Vogel) Koehne (Hatusima 1967) or *L.thunbergii* (DC.) Nakai (Ohashi et al. 2009)] and one species of a related genus, *Campylotropismacrocarpa* (Bunge) Rehder was sampled for outgroup comparison. The GenBank accession numbers are listed in Appendix I. Most sequences were downloaded from GenBank, except for the new species, which was newly sequenced in the present study. Three samples of the new species were sequenced and were identical, of which only one sequence (MZ468553) was selected for the phylogenetic analysis. Genomic DNA was extracted from silica-gel-dried leaves using the modified 2 × CTAB procedure of Doyle and Doyle (1987). The ITS sequences were amplified with primer pairs ITS4/ITSA, with PCR amplification and sequencing following Xu et al. (2012). The phylogenetic relationships were assessed using the Maximum Likelihood (ML) method, which was constructed using the programme IQ-TREE (Nguyen et al. 2015).

## Results

### Molecular phylogenetics

**Figure 2.**
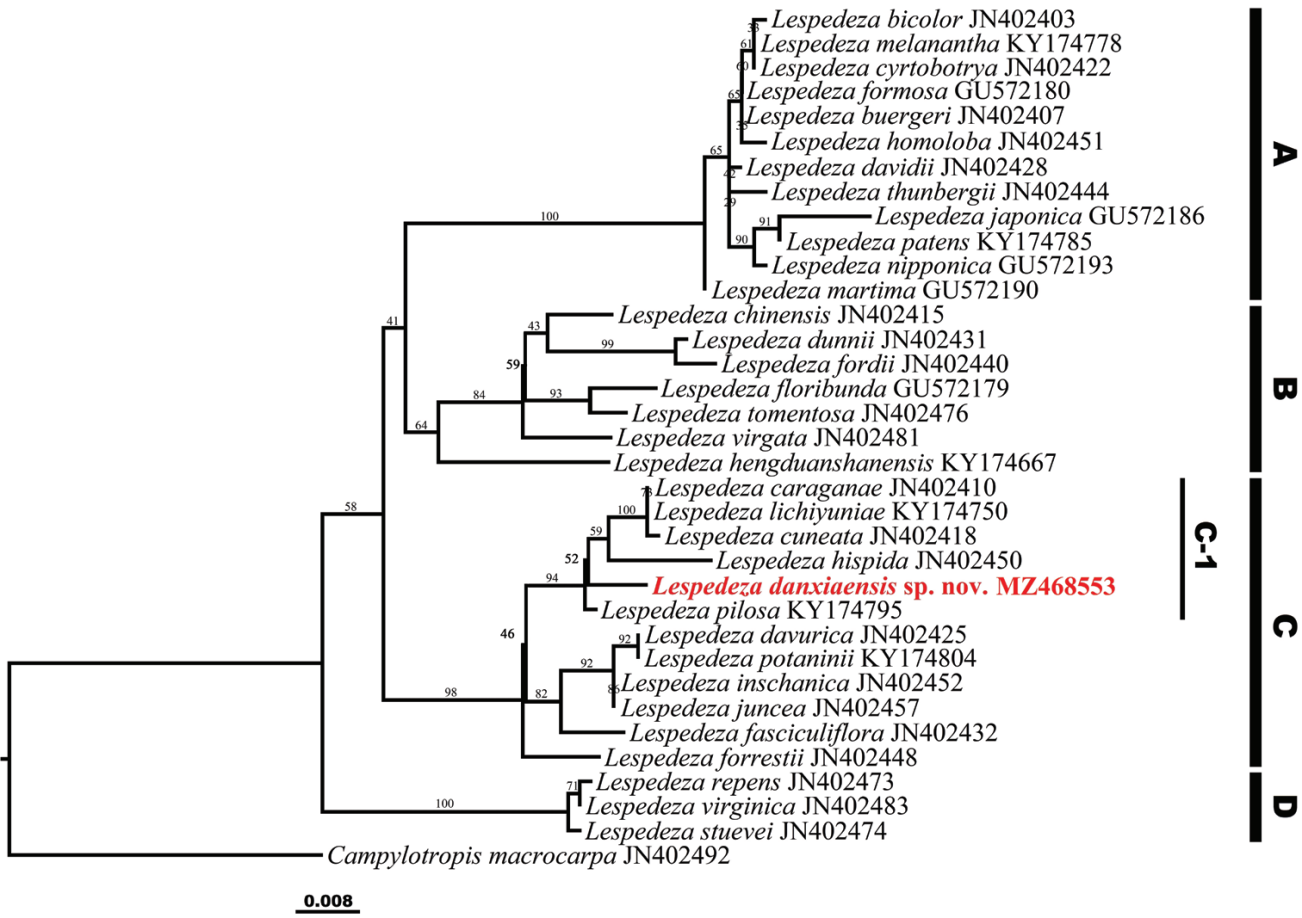
Phylogenetic relationships amongst 33 species of *Lespedeza* and *Campylotropismacrocarpa* based on ITS sequences using Maximum Likelihood analysis, bootstrap value of the Maximum Likelihood (LP) are shown along the branches. The new species described in this study is shown in bold and red type.

The aligned sequences of ITS for phylogenetic analyses are 702 bp in length. *Lespedeza* was recovered as monophyletic in the resulting phylogenetic tree in this study (LP: 100, Fig. 2). The North American *Lespedeza* taxa were clustered into a clade (clade D) as sister to the Asian taxa, of which were divided into three clades (viz. clade A, B and C) (LP: 100, Fig. 2). The putative new species is deeply nested within the clade C and was strongly supported as a member of subclade C-1 consisting of *L.caraganae* Bunge, *L.cuneata* G. Don, *L.hispida* (Franch.) T. Nemoto & H. Ohashi, *L.lichiyuniae* T. Nemoto, H. Ohashi & T. Itoh and *L.pilosa* (Thunb.) Siebold & Zucc. (LP = 94, Fig. 2).

### Morphological comparison

**Figure 3.**
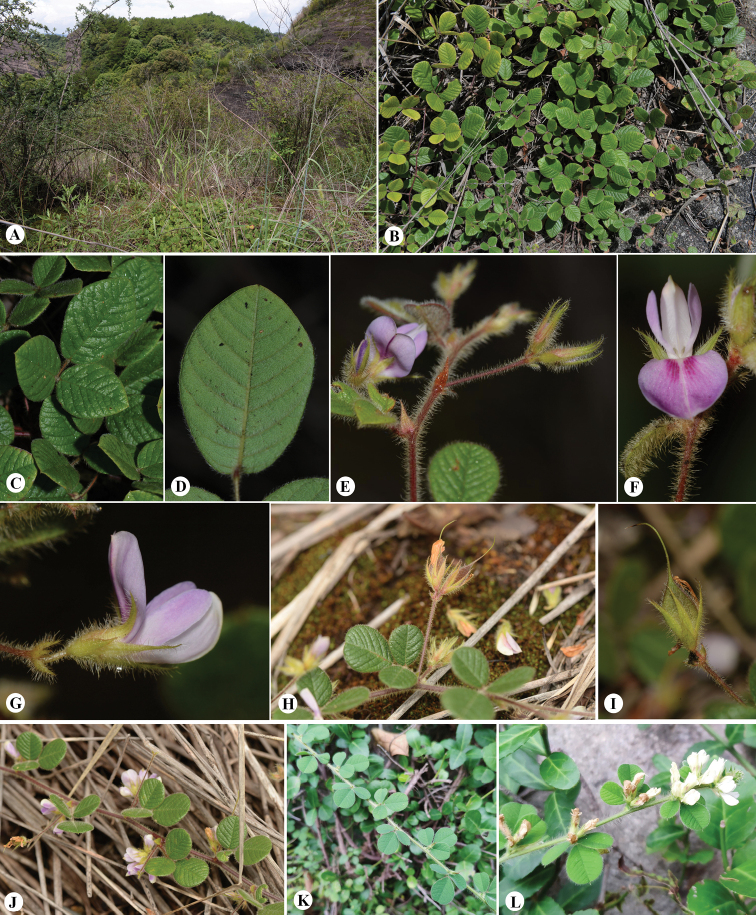
*Lespedezadanxiaensis* Q. Fan, W.Y. Zhao & K.W. Jiang and *L.pilosa* (Thunb.) Siebold & Zucc. *L.danxiaensis* (**A–J**) **A** habit, bushwood on the mountaintop of *Danxia* landform **B** plant, stems procumbent **C** adaxial view of leaf, surface green, leather **D** abaxial view of one leaflet, surface greyish-green with densely pubescent **E** flowering branchlet with flower bud, stipule triangular-lanceolate, apex acute **F** front view of flower **G** lateral view of flower, bracteoles long ovate, sepals narrowly lanceolate **H** fruiting branchlet, show the long peduncles **I** fruit, densely pubescent, stamens persistent **J** flowering branchlet, peduncles usually longer than 1 cm, flower pink to pale purple, young branch reddish brown *L.pilosa* (**K–L**) **K** branchlet with unripe fruit, leaf papery **L** flowering branchlet, peduncles short, flower white, young branch green. (Photographs: **A-J** by Qiang Fan **K–L** by Kai-Wen Jiang).

A detailed morphological comparisons of the new species with the five closely related species within subclade C-1 are summarized in Table 1. In morphology, the putative new species is most similar to *L.pilosa*, sharing such features as procumbent stem, ovate to obovate leaf blades, and plant covered densely villous indumentum. However, the new species differs from the latter by leathery leaflets, longer peduncles of chasmogamous flowers, and pink to pale purple corolla (Table 1, Fig. 3). The other four species included in subclade C-1 could be easily distinguishable from the new species by their habits (stem erect vs. stem procumbent), narrow leaf shape (oblong-linear to narrowly obovate leaf vs. ovate, obovate to subrounded), and shorter peduncles (0.5–1.0 mm vs. 11–28 mm) (Table 1).

**Table 1.** Morphological comparison of *Lespedezadanxiaensis* with its closest relatives.

**Characters**	***L.danxiaensis***	***L.pilosa***	***L.caraganae***	***L.cuneata***	***L.hispida***	***L.lichiyuniae***
Habit	Procumbent	Procumbent	Erect	Erect or ascending	Erect or ascending	Erect or ascending
Leaf texture	Leathery or thin leathery	Papery	Papery	Papery	Papery	Papery
Leaf shape	Ovate, obovate to subrounded	Broadly obovate or obovate	Oblong-linear	Cuneate or linear-cuneate	Narrowly obtriangular or narrowly obovate	Narrowly obovate
Adaxial surface of leaflet	Pubescent with ± adpressing hairs, more dense along the margin	White ascending-pilose	Subglabrous	Subglabrous	Glabrous	Glabrous
Abaxial surface of leaflet	Densely pubescent with ± adpressing hairs and more dense along the veins	White ascending-pilose	Adpressed hairy	Densely adpressed hairy	Densely adpressed or ascending pubescent	Densely appressed hairy
Peduncles of chasmogamous flowers (mm)	(2–) 11–28	0.5–1	0.5–1	Short	Ca. 1	Short
Flower color	Pink to pale purple	Yellowish white or white	White or yellow	Yellowish or white	White	Pink or pale purple

### Taxonomic treatment

#### Lespedeza danxiaensis

Taxon classificationPlantaeFabalesFabaceae

Q. Fan, W.Y. Zhao & K.W. Jiang sp. nov.

81D9E211-3443-583D-94A0-0728C38A2C69

urn:lsid:ipni.org:names:77222602-1

##### Type.

**Figure 4.**
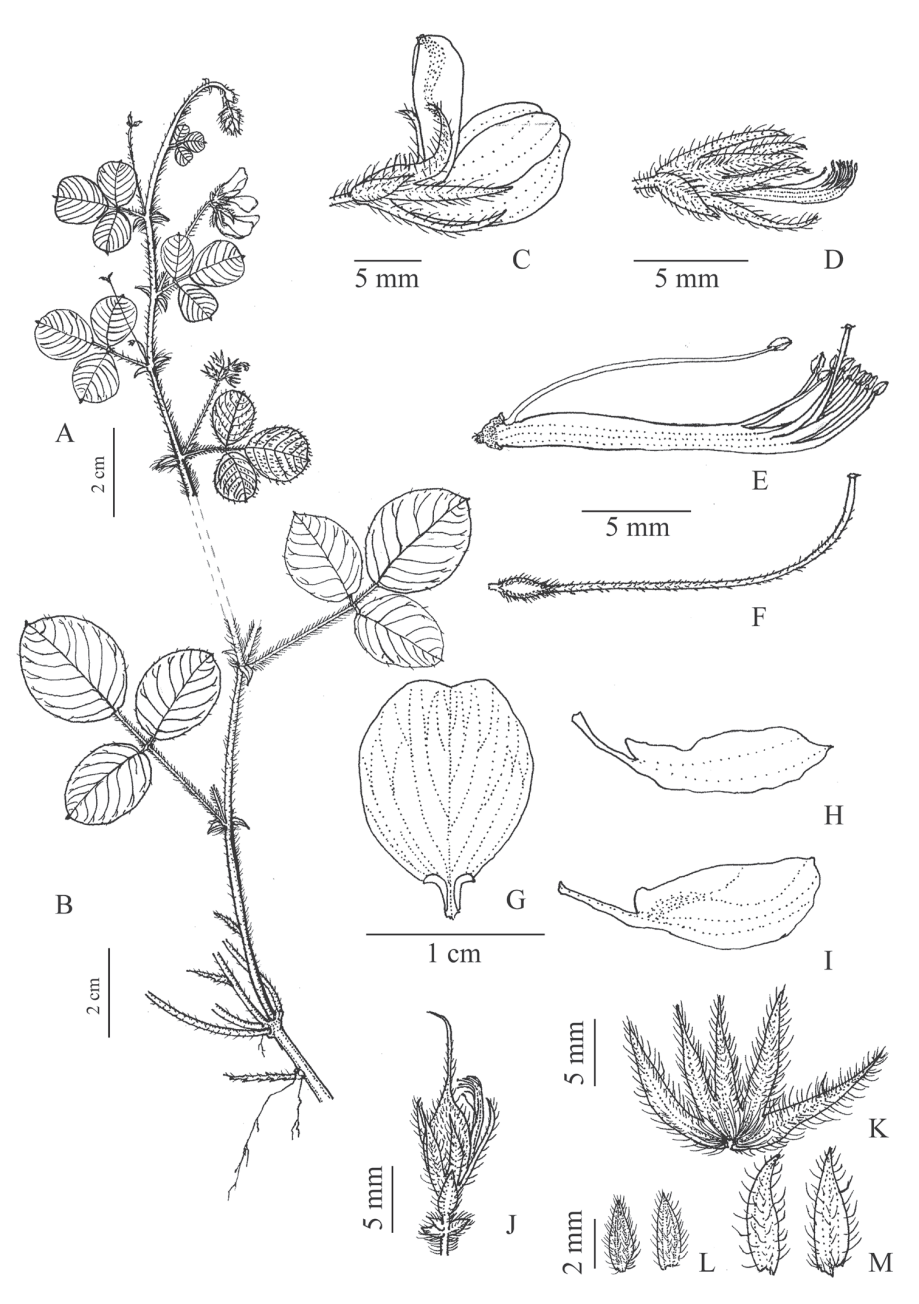
*Lespedezadanxiaensis* Q. Fan, W.Y. Zhao & K.W. Jiang **A** upper portion of plant **B** lower portion of plant **C** chasmogamous flower **D** cleistogamous flower **E** staminal tube **F** pistil **G** standard **H** wing-petal **I** keel-petal **J** chasmogamous fruit **K** abaxial view of calyx, flattened **L** bracts **M** bracteoles. (Drawn by Rong-En Wu).

China. Guangdong: Renhua County, Danxiashan National Nature Reserve, 24°56'N, 113°45'E, 290 m a.s.l., 30 Sept 2020, *Q. Fan 18409* (holotype, SYS!; isotypes IBSC!, NPH!, SWFC!, SYS!). (Figs 3, 4)

##### Diagnosis.

*L.danxiaensis* is most similar to *L.pilosa* morphologically both being densely villous throughout, and having procumbent stems with ovate to obovate leaflets, but differs from the latter by its leathery leaflets with obviously concave veins (vs. leaflets papery, veins slightly concave), pink to pale purple corolla (vs. corolla yellowish-white to white, with purple spots at base of the standard) and longer peduncles of chasmogamous flowers (1.1–2.8 cm vs. peduncles of chasmogamous flowers rather short, 0.5–1.0 mm in *L.pilosa*).

##### Description.

Perennial herbs, evergreen, with densely erect or ascending villous hairs throughout, turn sparse when old. **Stems** procumbent or ascending, woody at base, 50 cm tall. **Leaves** alternate, 3-foliolate; stipules persistent, ovate-triangular to triangular-lanceolate, apex acute, 3.5–4.5 mm, with 3–5 veins, sparsely pubescent; petioles 1.4–3.8 cm, densely pubescent; rachis 0.5–1.3 cm, densely pubescent, leaflets leathery, adaxially green, pubescent with ± adpressed hairs, more dense along the margin, abaxially greyish-green, more densely pubescent with ± adpressing hairs and more dense along the veins, lateral veins 8–12 pairs, obviously concave adaxially and prominent abaxially; terminal leaflets slightly larger than lateral ones, ovate to obovate, 2.2–3.8 × 1.5–2.5 cm, obtuse at apex, apiculate, rounded at base; lateral leaflets ovate to sub-rounded, 1.7–3.0 × 1.4–2.3 cm; petiolule ca. 1 mm; the leaves on flowering branches obviously smaller (with rachis 2–5 mm long; terminal leaflets obovate, 1.2–1.8 × 0.8–1.7 cm, apex obtuse or emarginate, broadly cuneate at base, lateral ones rounded to obovate, 0.9–1.5 × 0.7–1.2 cm). **Inflorescence** a pseudoraceme, 1–2 axillary, with 2–4 flowers per inflorescence, 2-flowered per node; peduncles of chasmogamous flowers slender and pubescent, (0.2–)1.1–2.8 cm, those of cleistogamous flowers reduced to 1–4 mm, on upper part of stems sometimes reduced; bracts 2 per node, narrowly ovate-triangular to broadly triangular, acute at apex, 1.5–3.3 mm, sparsely pubescent adaxially, glabrous abaxially, 3–5-veined. **Pedicel** 0.5–2.0 mm, pubescent; bracteoles 2, adnate to the base of the calyx, shorter than the calyx tube, oblong-ovate to ovate-lanceolate, 3.5–5.5 mm, sparsely pubescent, 5(–7)-veined. **Calyx** deeply 5-lobed almost to the base, densely pubescent adaxially, glabrous abaxially; tube ca. 1 mm; lobes lanceolate, sub-equal, 7–8 × ca. 1 mm, acute at apex. **Corolla** exserted (absent in cleistogamous flowers), pink to pale purple; standard pale purple, with dark purple spots at base, longer than wings and keels, inflexed-auriculate at base, lamina 7.5–8.0 × 6.5–7.0 mm, broadly elliptic to sub-orbicular, apex obtuse or emarginate, attenuate to a claw ca. 1 mm long at base; wings pale purplish-white, slightly shorter than keels, 7.5–8.3 mm with lamina 5.5–6.0 × 2.3–2.6 mm, narrowly ovate, obtuse at apex, slightly auriculate at base, with a basal claw ca. 2.5 mm; keel petals white to pale purplish-white, 7.5–8.5 mm with lamina 5.5–6.0 × 2.8–3.0 mm, obovate to elliptic, obtuse at apex, attenuate to a claw ca. 2.5 mm at base. **Stamens** glabrous, (9+1) diadelphous, ca. 9 mm, curved upwards in distal part; staminal tubes ca. 5 mm; anthers uniform, ovate, ca. 0.5 mm. **Pistils** ca. 10 mm, longer than stamens (shorter than stamens in cleistogamous flowers); ovary narrowly elliptic, shortly stipitate, style filamentous, curved upwards in distal part, ascending-pubescent; stigma terminal, capitate. **Pods** brownish, 1-seeded, elliptic, style persistent at apex, rostrate, 7–9 × ca. 3 mm, densely ascending-pubescent; those of cleistogamous flowers not seen. **Seeds** ovate, ca. 3.0 × ca. 1.4 mm.

##### Phenology.

Flowering from June to October, fruiting from September to December.

##### Etymology.

The specific epithet refers to Mount Danxia, the locality of the type collection. The Chinese name of the new species is here given as 丹霞铁马鞭 (Dān xiá

tiě mǎ biān), in which “丹霞” is the Chinese name for Mount Danxia, as well as “铁马鞭” being the common name for *Lespedezapilosa* and its allies.

##### Distribution, ecology and habitat.

*Lespedezadanxiaensis* is currently known only from a few populations on Mount Danxia in Renhua County, Guangdong Province of China. It was observed to occur in bushwood on the mountaintop of Danxia landform at elevations between 270 and 310 m; plants in association included *Osteomelessubrotunda* K. Koch, *Abeliachinensis* R. Br., *Lagerstroemiaindica* L., *Selaginellatamariscina* (P. Beauv.) Spring etc.

##### Conservation status.

The known localities of *Lespedezadanxiaensis* are in Danxiashan National Nature Reserve where they are well protected. However, its population size is quite small. There are fewer than 100 individuals surviving in an area of about 200 m^2^ in the currently known localities. We carried out several field surveys in 2020 from May to October, but no other populations were found. Due to its limited distributional range and small population size, *Lespedezadanxiaensis* is here recommended as **Critically Endangered (CR, B2a)** according to IUCN Categories (IUCN Standards and Petitions Subcommittee 2019).

##### Additional specimens examined (paratypes).

China. Guangdong: Renhua County, Danxiashan National Nature Reserve, 24°56'N, 113°45'E, 290 m a.s.l., 3 July 2020, *Q. Fan 18027* (IBSC, NPH, SWFC, SYS); *ibid*., 14 August 2020, *Q. Fan & Y. S. Huang 18130* (IBSC, NPH, SYS).

## Discussion

It is obvious that the new species belongs to *Lespedeza* due to its persistent bracts with two flowers inside, non-articulate pedicels, and 1-seeded pods (Fig. 3). Our molecular phylogenetic results further support the inclusion of the new species within Lespedezasubg.Macrolespedeza re-circumscribed by Ohashi and Nemoto (2014) (Fig. 2). The most conspicuous character of *L.danxiaensis* is its procumbent stems. There are only three procumbent *Lespedeza* species formerly recorded in China, i.e., *L.fasciculiflora*, *L.hengduanshanensis*, and *L.pilosa*. However, the former two species, occurring in western China (northwestern Yunnan, western Sichuan and Tibet) (Huang et al. 2010; Xu et al. 2014), are distantly related to the new species in the phylogenetic tree (Fig. 2). The third species *L.pilosa* is close to the new species, but they differ in the leaf texture, flower color, and the peduncle length of the chasmogamous flowers as described above. In addition, the ITS sequences of the three individuals of the new species are identical and no heterozygous sites were detected in these sequences, indicating that *L.danxiaensis* is not of hybrid origin, but a distinct species.

*Lespedezadanxiaensis* is current only known from the type locality, i.e. Mount Danxia, and only one population with fewer than 100 individuals was found by the authors. They grow in the special habitat of the Danxia landform, confined to the sub-top area of a peak. The special habitat may lead the phenomenon in which the number of this species is extremely small, thus the conservation of the species, including *ex situ* and *in situ* conservation, is urgently needed. *Lespedezadanxiaensis* has a procumbent habit, usually growing in patches on the ground, and is drought-tolerant. Our observations found that the above-ground part of the species survives drought by dropping many leaves during the dry season. Thus, this species may be suitable as a slope protection or soil-and-water conservation plant, which has potential development and application value.

## Supplementary Material

XML Treatment for Lespedeza danxiaensis

## Appendix I

**Table A1.** List of the GenBank accession numbers of the ITS sequences of sampled species in this study.

Species	GenBank Accession Number
*Campylotropismacrocarpa* (Bunge) Rehder	JN402492
*Lespedezabicolor* Turcz.	JN402403
*Lespedezabuergeri* Miq.	JN402407
*Lespedezacaraganae* Bunge	JN402410
*Lespedezachinensis* G. Don	JN402415
*Lespedezacuneata* G. Don	JN402418
*Lespedezacyrtobotrya* Miq.	JN402422
*Lespedezadanxiaensis* Q. Fan, W. Y. Zhao & K. W. Jiang	MZ468553 MZ468554 MZ468555
*Lespedezadavidii* Franch.	JN402428
*Lespedezadavurica* (Laxm.) Schindl.	JN402425
*Lespedezadunnii* Schindl.	JN402431
*Lespedezafasciculiflora* Franch.	JN402452
*Lespedezafloribunda* Bunge	GU572179
*Lespedezafordii* Schindl.	JN402440
*Lespedezaformosa* (Vogel) Koehne	GU572180
*Lespedezaforrestii* Schindl.	JN402448
*Lespedezafrutescens* (L.) Hornem.	JN402454
*Lespedezahengduanshanensis* (C.J. Chen) Bo Xu bis, X.F. Gao & Li Bing Zhang	KY174667
*Lespedezahirta* (L.) Hornem.	JN402449
*Lespedezahispida* (Franch.) T. Nemoto & H. Ohashi	JN402450
*Lespedezahomoloba* Nakai	JN402451
*Lespedezainschanica* Schindl.	JN402452
*Lespedezajaponica* L.H. Bailey	GU572186
*Lespedezajuncea* (L. f.) Pers.	JN402457
*Lespedezalichiyuniae* T. Nemoto, H. Ohashi & T. Itoh	KY174750
*Lespedezamaritima* Nakai	GU572190
*Lespedezamelanantha* Nakai	KY174778
*Lespedezanipponica* Nakai	GU572193
*Lespedezapatens* Nakai	KY174785
*Lespedezapilosa* Siebold & Zucc.	KY174795
*Lespedezapotaninii* V.N. Vassil.	KY174804
*Lespedezarepens* W.P.C. Barton	JN402473
*Lespedezastuevei* Nutt.	JN402474
*Lespedezathunbergii* (DC.) Nakai	GU572186
*Lespedezatomentosa* Siebold ex Maxim.	JN402476
*Lespedezavirgata* DC.	JN402481
*Lespedezavirginica* (L.) Britton	JN402483
